# Commentary: Transcranial stimulation of the frontal lobes increases propensity of mind-wandering without changing meta-awareness

**DOI:** 10.3389/fpsyg.2019.00130

**Published:** 2019-02-05

**Authors:** Gábor Csifcsák, Nya Mehnwolo Boayue, Per M. Aslaksen, Zsolt Turi, Andrea Antal, Josephine Groot, Guy E. Hawkins, Birte U. Forstmann, Alexander Opitz, Axel Thielscher, Matthias Mittner

**Affiliations:** ^1^Department of Psychology, UiT The Arctic University of Norway, Tromsø, Norway; ^2^Department of Clinical Neurophysiology, University Medical Center Goettingen, Göttingen, Germany; ^3^Integrative Model-based Cognitive Neuroscience Research Unit, University of Amsterdam, Amsterdam, Netherlands; ^4^School of Psychology, University of Newcastle, Newcastle, NSW, Australia; ^5^Department of Biomedical Engineering, University of Minnesota, Minneapolis, MN, United States; ^6^Danish Research Centre for Magnetic Resonance, Copenhagen University Hospital, Hvidovre, Denmark; ^7^Department of Electrical Engineering, Technical University of Denmark, Kongens Lyngby, Denmark

**Keywords:** mind wadering, transcranial direct cortical stimulation (tDCS), replication, dorsolateral preforntal cortex (DLPFC), Bayesian analysis

William James famously described attention as the process of “taking possession of the mind, in clear and vivid form, of one out of what seem several simultaneously possible objects or trains of thought” (James, 1890). Although put into words more than 125 years ago, this brilliant insight captures many aspects of human cognition that puzzle researchers even today. The process of how we select between different trains of thought and might become preoccupied with internally generated mental experiences is most commonly referred to as mind wandering (MW). Given that activity in the dorsolateral prefrontal cortex (DLPFC) has been linked to the onset and maintenance of MW episodes (Christoff et al., 2009; Smallwood et al., 2011; Mittner et al., 2014, 2016), non-invasive brain stimulation over that structure seems to be a suitable technique to interfere with MW. This approach can provide a causal link between DLPFC activity and MW, which in turn has the potential for developing new interventions to influence attentional lapses in health and disease (Berman et al., 2010; He et al., 2011; Bozhilova et al., 2018).

In 2015, a seminal study reported that anodal transcranial direct current stimulation (tDCS) above the left DLPFC could increase MW propensity in healthy adults (Axelrod et al., 2015). Participants were asked to perform the Sustained Attention to Response Task (SART), while MW was quantified using thought-probes both during and immediately following tDCS. The results of this study, however, were based on very small sample sizes (10–14 participants/group), and it is well-known that such underpowered studies necessarily overestimate effect sizes (Gelman and Carlin, 2014; Minarik et al., 2016). Indeed, Axelrod et al. (2015) reported a disproportionally large effect size of *d* = 1.24, which by far exceeds typical effect sizes in psychology. Given the current concerns about replicability in psychology (Open Science Collaboration, 2015) and tDCS in particular (Horvath et al., 2015), it is important to replicate the effect found by Axelrod et al. (2015) before drawing firm conclusions. Recently, the first author of the original study reported a successful replication (Axelrod et al., 2018) with the inclusion of a larger sample size (27–30 participants/group) obtaining an effect size of a similar magnitude, *d* = 0.97. Moreover, the new study was conducted in a different country (China instead of Israel) and language. This second study seems to provide converging evidence for the utility of anodal tDCS above the DLPFC in increasing MW propensity.

Based on our team's own work, here we critically discuss and evaluate concerns about the replicability of the reported effects. When the original study was published, we initiated a pre-registered, multi-site, high-powered replication attempt of that study (Axelrod et al., 2015). Pre-registration is considered to be the best way to perform objective, rigorous, and fully transparent data analysis, to avoid publication bias and thus, to promote publishing research with high replicability (Chambers et al., 2014). In the planning phase we contacted the original authors to ensure that our protocol matched the original protocol as closely as possible. We used Bayesian design analysis so we could quantify both the evidence for or against the efficacy of anodal tDCS relative to sham stimulation in modulating MW, and to provide a more reasonable estimate for the putative effect size (Boayue et al., 2018). The analysis and data-collection plan was pre-registered and accepted at the European Journal of Neuroscience. Subsequently, we collected data in three independent laboratories and across three languages (Dutch, German and Norwegian), with a total of 192 participants. Data analysis was performed in accordance with the pre-registered plan, and all scripts and raw data are available at https://osf.io/dct2r/. Our primary result was evidence for the absence of a facilitatory effect of tDCS on MW propensity. In fact, we even found numerically larger MW scores for sham tDCS, with an effect size estimate of *d* = −0.11 (although the posterior highest density interval included zero, indicating that this effect may not be robust). A Bayesian measure specifically designed to test replication success (Verhagen and Wagenmakers, 2014) suggested that it is about 500 times more likely that the effect did not replicate (vs. the effect replicated). This was also confirmed by the Bayes Factor quantifying evidence for a null effect (with a value of 10.65), indicating strong evidence against the existence of the effect. These findings were consistent across the three laboratories (Boayue et al., 2018).

Our findings are in stark contrast to (Axelrod et al., 2018) replication attempt. We also note that our study protocol was actually more similar to the original study (Axelrod et al., 2015) than the one used by Axelrod et al. (2018) in their own subsequent replication. For instance, we closely complied with all aspects of the tDCS protocol (except that we implemented a more thorough double-blinding procedure), whereas Axelrod et al. (2018) used longer stimulation times (30 min instead of 20 min) and a larger electrode size above the target region (5 x 7 cm instead of 4 x 4 cm). The functional relevance of these changes for any effect on MW is not clear, but the electric field induced in the brain can vary widely (Csifcsák et al., 2018). Due to the inconsistent use of stimulation parameters, the recent publication by Axelrod et al. (2018) can hardly be regarded a direct replication of the original study. Moreover, the sample size calculation of their replication attempt was based on the originally reported effect size (*d* = 1), which, as we detailed above, is extremely likely to be overestimated. In this respect, although the estimated sample size of 26 participants/stimulation protocol was met by Axelrod et al. (2018), a more realistic effect size of around 0.4 estimated in tDCS studies (Horvath et al., 2015; Minarik et al., 2016) would require at least 78 participants/group (one-tailed *t*-test, alpha = 0.05, power = 0.8), which criterion was fulfilled only by our study (96 participants/group). Given that our study design and analysis plan was peer reviewed and pre-registered prior to data collection, was conducted in three independent laboratories, and featured a much larger sample size, we suggest here that it is more likely that the putative effect does not exist.

We believe that one path toward a more detailed perspective on this issue is to perform a meta-analysis across all available datasets so far (we are not aware of any other replication attempts). This analysis would be based on data from a total of 270 participants speaking 5 different languages, recruited from 5 independent laboratories located in different countries. To date, our attempts to obtain the raw data from either of the studies conducted by Axelrod and colleagues have not been fruitful. Given this state of affairs, we therefore conclude that the enthusiasm about the finding by Axelrod et al. (2015) seems to have been premature.

## Author Contributions

All authors listed have made a substantial, direct and intellectual contribution to the work, and approved it for publication.

### Conflict of Interest Statement

The authors declare that the research was conducted in the absence of any commercial or financial relationships that could be construed as a potential conflict of interest.
